# Supplementary material for: High-precision tracking and positioning for monitoring Holstein cattle
Source: PLoS One. 2024 May 14;19(5):e0302277. doi: 10.1371/journal.pone.0302277 (PMC11093326; doi:10.1371/journal.pone.0302277)
Supplement: S1 Table — (PDF) [file pone.0302277.s001.pdf]

S1 Table. Hardware parameters:

Hardware Name	Specific parameter settings
1. Rack	Aircraft type: quadcopter<br>Weight (approximately): 2.2 kg (excluding battery, including load), 3.2 kg (including battery, including load)<br>Diagonal wheelbase: 600 mm<br>Power system: 6S FOC governor + 4006 motor + 370 mm<br>Maximum payload of blade: 0.8 kg (+ pod + LiDAR)<br>Maximum takeoff weight: 4 kg<br>Maximum flight time: 36 min 22 s (high configuration, 4 m flight altitude, outdoor 19 degrees)<br>Hover accuracy: 0.5 m vertically and 1.5 m horizontally<br>Wind resistance level: 6 - 7<br>Working environment: outdoor<br>Working temperature: 6℃ - 40℃
2. Built in flight control	Flight control kernel: Based on Pixhawk FMUv5 deep optimization design, 90% compatible with PX4 FMUv5.<br>Integrated distribution board: DC-DC/2-ch 12 V 3A/1-ch 5 V 3A/4 * XT30 interface/100 A solid-state relay<br>Main MCU chip: STM32F765VI/216MHz/2M program memory/512KB data memory<br>Gyroscope: ICM20689<br>Barometer: BMP388<br>MTD：AT24C64
3. External expansion platform	Power interface: 1 * XT30 battery voltage power supply port<br>I/O interface: built-in PX4IO-V2, supporting 8 * PWM interface/1 * RC interface (supporting Sbus, PPM, DSM)<br>External interface: 3 * UART (GH1.25 6 Pin)/1 * CAN (GH1.25 4 Pin)/Flight control USB connection (Type-C)
4. On board computer	Name: Allspark<br>Model: AS1X

	Weight: 213 g<br>Size: 94 * 59 * 37 mm<br>Processor: NVIDIA Jetson NX<br>Memory：8G LPDDR4x @1600MHz<br>Emmc: 16 GB<br>SD card: 64 GB (maximum 128 GB)<br>Display: Micro HDMI (1,920 * 1,080 P)<br>Camera interface: MIPI Camera ×2<br>Ethernet port: 1,000 Mbps (adapter)<br>WiFi：2.4 G<br>USB interface: USB3.0 Port (Type A) ×2，USB3.0 Port (Type C) ×1，USB2.0 Port (Micro B)<br>×1 (OTG)<br>GPIO: GPIO × 5 (3.3 V)<br>CAN: CAN × 1 (3.3 V)<br>UART*3: UART × 3 (3.3 V, including Debug UART)<br>SPI: SPI × 1 (3.3 V)<br>Overall power: 6 - 24 W<br>Power input: 9 – 20 V (recommended 12 V, 2.5 A)<br>Working environment temperature: -20℃ - 50℃
5. Battery	Model: 6S-10000 mah lithium polymer battery<br>Effective capacity: 10,000 mAh<br>Battery weight: 1,008 g (approximately 1 kg)<br>Operating voltage: 22.2 - 26.1 V<br>Minimum voltage: 21 V (theoretical minimum 18 V, actual minimum 22 V)<br>Storage voltage: 23.1 V (stored according to a single section voltage of 3.85 V)<br>Battery interface: XT60<br>Battery type: LIHV lithium battery<br>Working temperature: 0℃ - 60℃
6. Remote control	Model: H16 Number of channels: 16<br>Frequency range: 2.400 - 2.483 GHz<br>RF power: 20DB@CE/23DB@FCC

	Frequency hopping: New FHSS frequency hopping<br>Working voltage: 4.2 V<br>Battery: 20,000 mAh<br>Endurance: 6 - 20 hours<br>Charging interface: Type-C<br>Upgrade: APP online upgrade<br>Size: 272 * 183 * 94 mm<br>Weight: 1,034 g
7. Communication link	Model: R16<br>Communication distance: 5 – 10 km (nominal), 3 km (measured in urban environment)<br>Bandwidth: 20 Mbps<br>Number of channels: 16<br>RF power: 20DB@CE/23DB@FCC<br>Working voltage: 7.2 - 72 V<br>Interface: MIPI input * 1; HDMI input * 1; Network port * 1; BAT+SBUS * 1 Type-C * 1; TF card slot; Serial port * 2<br>Size: 76 * 59 * 11 mm<br>Weight: 90 g
8. Searchlight camera	Model: MIPI ThreadBody<br>Video format: 1,920 * 1,080 @ 25 fps<br>Light effect component: 8 W high-power LED, 150 ° lens<br>Working voltage: 14 - 72 V<br>Working current: 14 mA (without turning on the light); 140 mA (with lights on)<br>Size: 105 * 43 * 35 mm<br>Weight: 55 g
9. Photoelectric pod	Model: Q10F Weight: 409 g<br>Dimensions: length 105 × width 91 × height 98 mm<br>Input voltage: 3 - 4 s<br>Video output: 360 P (30 fps), 480 P (30 fps, recommended for visual recognition) 720 P (10 fps), 1,080 P (5 fps)<br>Camera performance: 1/3 inch 4

	megapixel COMS SENSOR<br>Camera focal length: 10× optical zoom, F = 4.9 – 49 mm<br>Temperature working range: -10 - 45 degrees Celsius<br>Deviation pixel update rate: 50 Hz<br>Minimum target size: 16 × 16 pixels<br>Target locking steady-state error: < 5 pixels<br>Output interface: USB interface
10. LiDAR	Model: LDS-50C<br>Laser ranging technology: TOF<br>Measurement radius: 0.15 - 40 m<br>Sampling speed: 9,200 times/second<br>Distance measurement accuracy: 2 - 10 cm (typical value 5 cm)<br>Ranging resolution: 1 cm<br>Scanning angle: 360 °<br>Scanning frequency: 7 - 15 Hz (typical value 10 Hz)
