# Supplementary material for: High-precision tracking and positioning for monitoring Holstein cattle
Source: PLoS One. 2024 May 14;19(5):e0302277. doi: 10.1371/journal.pone.0302277 (PMC11093326; doi:10.1371/journal.pone.0302277)
Supplement: S2 Table — (PDF) [file pone.0302277.s002.pdf]

S2 Table. Algorithm

Algorithm 1 Two-stage matching algorithm

Input: the track indices in the previous frame $T = \{1, \ldots, N\}$, the detection indices $O = \{1, \ldots, M\}$ sorted by their confidence scores, Stranded area $S = \{\ldots\}$ initialized with the detections of the very first frame, the life values $L = \{l_i\}$, motion vectors toward future frames $V = \{v_i\}$, and center points of objects $C = \{c_i\}$.

Output: matching result A.

Initializing set of matches $A = \varnothing$
Computing the distance matrix D_1 between the tracks and the detections using Greedy Matching Algorithm Based on Distance
For i=1 to min(N,M)
  $j = \arg\min_k D_1(i,k)$
  If $D_1(i,j) \neq \infty$
    $A = A \cup \{(i,j)\}$
    $T = T - \{j\}$
    $O = O - \{i\}$
  End If
End For
If $O \neq \varnothing$
  Computing the distance matrix D_2 between the objects in the stranded area and the and the detections in O using Greedy Matching Algorithm Based on Distance
  For i in O
    $j = \arg\min_k D_2(i,k)$
    If $D_2(i,j) \neq \infty$
      $A = A \cup \{(i,j)\}$
      $O = O - \{i\}$
      $S = S - \{j\}$
    End If
  End For
End If
Updating the life values of objects in the stranded area: $l_i = l_i - 1$
Removing the objects from S if the life value is zero.
Adding the unmatched tracks into the stranded area and initialize their life value:
  $S = S \cup T, l_i = L_{\max} \; for\, i \in T$
Updating the locations of the objects in $S : c_i = c_i + v_i$
Return A
