# Supplementary material for: High-precision tracking and positioning for monitoring Holstein cattle
Source: PLoS One. 2024 May 14;19(5):e0302277. doi: 10.1371/journal.pone.0302277 (PMC11093326; doi:10.1371/journal.pone.0302277)
Supplement: Attachment — Submitted filename: renamed_245e0.docx [file pone.0302277.s004.docx]

This paper presents a method for using unmanned aerial vehicles (UAVs) to achieve multi-target tracking of cattle. Compared with the traditional tracking methods, the performance of the model has been significantly improved in all aspects.

This article has clear ideas, compact structure and beautiful and concise diagrams. However, the author must consider the following suggestions to further improve the quality of his work.

1. In the description of the current state of research, the existing research status should be described in more detail so that the reader can understand the advantages and disadvantages of other algorithms.
2. In the part of model design, I think the author should elaborate in more detail on the reasons for choosing various optimization strategies and whether this strategy has been applied on other models.
3. The author can add some pictures in the necessary places in the article to make the expression more intuitive and easy to understand.
