# Supplementary material for: High-precision tracking and positioning for monitoring Holstein cattle
Source: PLoS One. 2024 May 14;19(5):e0302277. doi: 10.1371/journal.pone.0302277 (PMC11093326; doi:10.1371/journal.pone.0302277)
Supplement: Attachment — Submitted filename: Response to Reviewers.docx [file pone.0302277.s005.docx]

**Additional Editor Comments:**

This paper introduces a UAV-based approach for multi-target tracking of cattle, demonstrating substantial improvements over traditional methods. The content is well-structured, featuring clear ideas and concise diagrams. However, the author is advised to enhance the paper&#39;s quality by providing a more detailed overview of existing research, outlining the strengths and weaknesses of other algorithms. Additionally, in the model design section, a more in-depth explanation of the rationale behind selecting optimization strategies and their application to other models is recommended.

Response:

Dear editor

Thank you so much for taking care of our study, and we have carefully revised the manuscript with comprehensive comments from all reviewers. We have incorporated these changes in the revision with YELLOW fonts representing revisions. We like to thank them for their significant contributions to our manuscript.

Thank you again for everything you have contributed, and I look forward to your approval.

Yours sincerely,

Wei Luo

Thank you very much for your suggestion, we have followed your suggestion to describe the existing research status in more detail in the "Introduction" section of the revised paper, and to describe the advantages and disadvantages of other algorithms in more detail. In addition, as you suggested, we have included in the Model Design section of the revised paper a more detailed description of the reasons for choosing the various optimization strategies, and whether such strategies have been applied to other models (we have cited specific references). Again, thank you for your suggestions, which have further improved the quality of our paper.

Review Comments to the Author

Please use the space provided to explain your answers to the questions above. You may also include additional comments for the author, including concerns about dual publication, research ethics, or publication ethics. (Please upload your review as an attachment if it exceeds 20,000 characters)

Reviewer #1: The paper is well written. The conclusion section needs to be improved. It should give more insight inot tthe implications of the results and should not duel on summarising the work done.
There is little or no discussion on future work and future directions of the work done. What are the implications? Are there any insights the authors wish to share?

Response: We are pleased to receive your suggestions for revisions. We are writing this response letter to explain the changes we made to the manuscript after discussion among all the authors. We have incorporated these changes into the revised manuscript. We would like to thank you for your important contribution to our manuscript. Please find below our detailed response to all of these comments/suggestions. Thank you again for all your contributions, and I look forward to your endorsement.

Thank you very much for your suggestions. We have revised the conclusion section in the revised draft according to your suggestions. We have simplified the summary of our work and focused on discussing the meaning of the results. In addition, at the end of the conclusion of the paper, we further discussed future work. Thank you again for your suggestions, they have greatly benefited us.

Reviewer #2: This paper presents a method for using unmanned aerial vehicles (UAVs) to achieve multi-target tracking of cattle. Compared with the traditional tracking methods, the performance of the model has been significantly improved in all aspects.
This article has clear ideas, compact structure and beautiful and concise diagrams. However, the author must consider the following suggestions to further improve the quality of his work.
Response: We are pleased to receive your suggestions for revisions. We are writing this response letter to explain the changes we made to the manuscript after discussion among all the authors. We have incorporated these changes into the revised manuscript. We would like to thank you for your important contribution to our manuscript. Please find below our detailed response to all of these comments/suggestions. Thank you again for all your contributions, and I look forward to your endorsement.

1.In the description of the current state of research, the existing research status should be described in more detail so that the reader can understand the advantages and disadvantages of other algorithms.

Response: Thank you very much for your suggestion. We have followed your advice and provided a more detailed description of the current research status and the advantages and disadvantages of other algorithms in the "Introduction" section of the revised paper. Once again, thank you for your suggestions. Your suggestions have further improved the quality of our paper.

2.In the part of model design, I think the author should elaborate in more detail on the reasons for choosing various optimization strategies and whether this strategy has been applied on other models.
Response: Thank you very much for your suggestion. We have followed your advice and elaborated in more detail on the reasons for choosing various optimization strategies in the model design section of the revised paper, as well as whether this strategy has been applied to other models(We have cited specific references). Once again, thank you for your suggestions. Your suggestions have further improved the quality of our paper.
3.The author can add some pictures in the necessary places in the article to make the expression more intuitive and easy to understand.

Response: Thank you very much for your suggestion. We have added Figure 7 in the target localization algorithm section of the revised draft to better illustrate the position of the drone relative to the target, thus better explaining the importance of target localization and making the expression more intuitive and understandable.

Reviewer #3: The paper falls into the class of Multi-Object-Tracking (MOT) systems for monitoring Holstein cattle. Here are my comments:

Response: We are pleased to receive your suggestions for revisions. We are writing this response letter to explain the changes we made to the manuscript after discussion among all the authors. We have incorporated these changes into the revised manuscript. We would like to thank you for your important contribution to our manuscript. Please find below our detailed response to all of these comments/suggestions. Thank you again for all your contributions, and I look forward to your endorsement.

1. The English should be proofread. I suggest changing the title of the paper to "High-precision tracking and positioning for monitoring Holstein cattle".

Response: Thank you very much for your suggestion. We have changed the title of our paper according to your suggestion, which can better summarize the content of our research. In addition, we have proofread the English of the paper to reduce grammar errors. Finally, thank you again for your suggestions. Your suggestions have greatly benefited us.
2. The experimental section should be expanded by providing (a) processing time of the approach in terms of FPS; (b) results on MOT71 and MOT20 benchmarks to get a full idea of the general performance of the proposed approach vs. competitors.

Response: Thank you very much for your suggestion. We have added FPS indicators for different methods in Table 3 of the revised manuscript as per your suggestion. In addition, we have added Table 4 as the results of the MOT17 and MOT20 benchmark tests to comprehensively demonstrate the overall performance of the proposed method compared to other methods. Finally, thank you again for your suggestions, which have further improved the quality of our paper.

Reviewer #4: Precision livestock farming utilises information technology to continuously monitor and manage livestock in real-time, which can improve individual animal health, welfare, productivity and the environmental impact of animal husbandry, contributing to the economic, social and environmental sustainability of livestock farming. Based on artificial intelligence, it promises to driving an agricultural revolution at a time when the world must produce more food using fewer resources.
Thus, Enhanced animal welfare emerges as a pivotal element in contemporary precision animal husbandry, with bovine monitoring constituting a significant facet of precision agriculture. Also, smart drones, outfitted with monitoring systems, have evolved into a viable solution for wildlife protection, moni-toring, and animal husbandry.
It is in this context that in this manuscript the authors propose a tracking algorithm, grounded in deep learning, adhering to the JDT paradigm estab-lished on the CenterTrack algorithm, designed to satisfy the requirements of mul-ti-objective tracking amidst intricate scenarios in practical applications.
The authors believe that the proposed algorithm achieved higher preformances compared to others known in the literature.
The manuscript needs much improvement in order to be accepted for publication. Authors should consider the comments/requirements/questions below:

Response: We are pleased to receive your suggestions for revisions. We are writing this response letter to explain the changes we made to the manuscript after discussion among all the authors. We have incorporated these changes into the revised manuscript. We would like to thank you for your important contribution to our manuscript. Please find below our detailed response to all of these comments/suggestions. Thank you again for all your contributions, and I look forward to your endorsement.
1). Remarks and suggestions:
* The introduction is nevertheless long, it contains a few sentences that can be skipped.
Response: Thank you very much for your suggestion. We have carefully checked the introduction of our paper and found some redundant sentences. We have reorganized the introduction in the revised draft and removed some redundant sentences and paragraphs. Finally, thank you again for your suggestions. Your suggestions have further improved the quality of our paper.
* Figures 1, a and b) are not clear, they need to be improved in quality.
Response: Thank you very much for your suggestion. We have revised Figure 1 in the revised draft. Due to the need for copyright proof for Figure 1 (a, b) in PLOS ONE journal, we reluctantly deleted Figure 1 (a, b), but introduced the latitude and longitude of the region in the paper. Replace Figure 1 (c, d) with clearer images to better showcase the research objectives.
* The result of the simulation as well as the performance measurements presented in the manuscript must be clearly commented and analyzed. The authors presented surface and generic discussions.
Response: Thank you very much for your suggestions. Based on your suggestions, we have conducted a clear analysis of the performance measurements in the revised draft. In order to facilitate readers to read more intuitively, we have placed this section (analysis from Tables 1 to 4) in the "Discussion" section. Finally, thank you again for your suggestions. Your suggestions have further improved the quality of our paper.
2). Questions
Q1 : The authors state the following sentences (page 13):
"The proposed two-stage matching algorithm consists of two matching stages, both of which ............... thereby preserving the continuity of the tracking trajectory. This process is depicted in Figure 7. "
How did you take this into your program?
Response: Thank you very much for your question. The baseline algorithm CenterTrack in this article uses a distance based greedy matching algorithm to achieve this process, but its effect in the face of occlusion is not ideal. Therefore, we added our proposed two-stage matching algorithm to the data association section of the program and directly replaced the distance based greedy matching algorithm in the original program (our two-stage matching algorithm is also equivalent to performing the distance based greedy matching algorithm twice). The pseudocode of the two-stage matching algorithm is shown in Appendix B.
Q2 : How can you reduce the likelihood of tracking failures and increase robustness in various lighting conditions?
Response: Thank you very much for your question, which was mentioned in our future work on the paper. In future work, we will integrate visible and infrared images and use the RGB-T algorithm with fusion attention mechanism for tracking under various lighting conditions to solve the challenge of a single sensor being unable to obtain accurate information due to lighting issues in complex environments such as low light and strong light, thereby reducing the possibility of tracking faults and improving robustness. We have added this part of the content to the "Conclusion" section of the revised manuscript for future work.
Q3 : How will you improve the accuracy in the MOT algorithm?

Response: Thank you very much for your question, which was mentioned in our future work on the paper. In future work, we will explore integrating global motion compensation into the MOT algorithm, which we consider as an image registration method suitable for revealing background motion. This method extracts image key points and sparse optical flow for feature tracking based on translational local anomaly suppression, thereby compensating for the motion of drone cameras and improving the accuracy of the MOT algorithm.
